# Bioadhesive Gauze Embedded with Chitosan-Butein Bioconjugate: A Redox-Active pH Sensor Platform

**DOI:** 10.3390/bios13010006

**Published:** 2022-12-21

**Authors:** Vinoth Krishnan, Venkatachalam Ananth, Jayasudha Velayutham, Pandiaraj Manickam, Murugan Veerapandian

**Affiliations:** 1 Electrodics and Electrocatalysis Division, CSIR-Central Electrochemical Research Institute (CECRI), Karaikudi, Sivagangai 630 003, TN, India; 2 Academy of Scientific & Innovative Research (AcSIR), Ghaziabad 201 002, UP, India

**Keywords:** flexible bandage electrode, functionalized biopolymer, wound pH sensor, 2D electrode fabrication, redox-active

## Abstract

With the ever-growing global wound care market, demand for robust redox-active healthcare material is obvious for the construction of wearable sensor platforms. Surface reactive functional group-rich material like chitosan holds huge potential for electrochemical biosensor application. Herein, a metal-free redox-active chitosan–butein (CSB) bioconjugate is processed into epidermal bioadhesive electrode material useful for pH sensors promising toward wound site analysis. A two-electrode system devised for conducting carbon-reinforced silver chloride paste and CSB-modified carbon/silver chloride matrix was used as a reference and working electrodes, respectively. Dimensions of working and reference electrodes (4 mm) were designed by 2D cutter plotter-assisted stenciling. The cross-sectional topology of the constructed adhesive CSB-sensor platform exhibits an average surface thickness of 183 ± 2 μm. Cyclic voltammetric analysis revealed the inherent 2e^−^/2H^+^ transfer attributed to the catechol OH groups of graft polymerized CSB modified on adhesive gauze. As-fabricated modified electrode substrates exhibit distinguishable potential differences with respect to electrolytes of varied pH (between 5 to 9), promising for wound site analysis.

## 1. Introduction

Recent advancements in the development of wearable devices on flexible polymeric substrates, papers, and textiles through various methods, including 3D printing, direct writing, ink-jet printing, and stencil printing, have attracted numerous healthcare applications, viz., electrocardiography (ECG), body fluid measurement, and wound healing analysis [1,2,3,4,5,6]. However, the challenge involved in the development of flexible electronics is the choice of conductive and biocompatible electrode ink. The conductive inks used for printable electronics should possess unique properties such as homogeneity in film formation, stability, and processability. Most of the attempted work on flexible sensor fabrication was focused on carbon-based conductive ink because of their electrical, mechanical, and robustness in addition to cost efficiency, compared to transition metal dichalcogenides (TMDs), black phosphorus (BP), hexagonal boron nitride (h-BN) and Dawson type heteropolyanions [7,8,9,10].

For instance, carbon nanotubes (CNT), including single-/multi-walled and graphene-based conductive ink, were processed for various flexible and optoelectronic devices [11,12]. In addition, with the rapid development of wearable electronics and electrical biosensors, the biocompatibility and biodegradability of the electrode ink are also of major concern in biomedical research. Recent government policies on bio-/eco-friendly approaches urged a huge demand for multifunctional material development towards healthcare applications. In this context, researchers are moving towards polymer-based materials which possess low toxicity, biocompatibility, cost efficiency, and scalability for repair and regeneration applications [13]. A wide range of polymers, including natural, synthetic, and hybrid conducting polymers, are identified as potential candidates for biomedical applications. Amongst, the naturally derived biopolymers have excellent biological properties for interdisciplinary applications. However, the poor electronic and mechanical properties of biopolymers may lack utility in wearable electronics development [14,15]. The other two polymeric materials were reasonably explored in various tissue engineering applications. For instance, a three-dimensional (3D) printing of polycaprolactone (PCL) incorporated with CNT and iron oxide nanocomposite for cardiac and bone tissue engineering applications, respectively [16,17]. Similarly, reports were also found on the demonstration of conducting polymers towards biomedical applications, viz., poly-aniline doped bacterial cellulose conductive nanocomposite membrane for flexible electronics [18] and pyrrole-containing nanofibrous membrane for cardiac tissue engineering applications [19]. Even though superior conductivity is endowed for bioelectronics development using synthetic and conductive polymers, the poor biological and mechanical properties lack its usage in biomedical applications. As mentioned above, over the years, the biopolymer derivative has shown a greater interest in interdisciplinary applications. Amongst the naturally abundant biopolymer, chitosan (CS) is a linear polysaccharide produced through the deacetylation of chitin, which also has a potential market value and broader scope in healthcare and environmental applications [20]. CS is non-toxic and biodegradable; further, the key characteristics such as electronic conductivity and mechanical property open up their utility in multiple sectors. It is hypothesized that the functional surface of CS can be tuned with other electroactive materials for wearable/flexible electronics.

For scalable point-of-care technology and eco-friendly application, metal-free electrodes are regarded as the optimal choice of interest. Considering the abundance of cost-efficient natural product-derived precursor sources, in recent days, significant efforts have been devoted to processing metal-free components like polyphenol/MWCNT, carbon nanoparticles from waste, and graphene derivatives for electrode materials [21,22,23]. Similar to conducting polymers, selected polyphenols are electroactive and bio-functional suitable for biomedical theranostics [24]. The existing functional group on the aromatic/heterocyclic ring structure determine the optical and electrochemical property of polyphenolic phytochemical products [25]. For instance, butein (B) is a chalcone-type flavonoid, ((E)-1-(2′, 4′ -dihydroxyphenyl)-3-(3,4-dihydroxyphenyl) prop-2-en-1-one), known for anti-inflammatory, anti-cancer, and anti-bacterial properties. An electroactive catecholic OH group in butein is known for the electron transfer reactions at the interfaces. Recently, our group prepared the CSB bioconjugate via the graft polymerization technique [26], as the prepared material exhibits water dispersibility, biocompatibility, biodegradability, and suitable rheology for versatile applications in biosystems. Herein, the CSB bioconjugate is processed on the adhesive pads for the first time to monitor the pH changes promising toward the wound site analysis. Wounds are a debilitating disorder that causes severe grief to annoyed patients. The current method of screening the wound healing process/markers relies on enzyme-linked immunosorbent assays (ELISAs) [27]. Some of the wound site markers include cytokines, microorganisms, and exudates pH [5]. Thus, the development of point-of-care testing could be advantageous for monitoring the chronic wound healing status, especially in geriatrics with diabetic foot, venous, and pressure ulcer diseases [28]. In that aspect, herein, 2D stencil-assisted conductive tracks were devised by conductive carbon-reinforced silver chloride (AgCl) ink on pre-treated adhesive pads and integrated with various wound dressings. Scanning electron microscope (SEM) and contact angle measurement evidenced the uniform surface morphology, coating thickness, and wettability nature of the electrode, respectively. Cyclic voltammetric (CV) analysis reveals the inherent redox behavior of CSB at the electrode-electrolyte interface against a wide pH range, promising toward wound site analysis.

## 2. Materials and Methods

### 2.1. Chemicals and Reagents

2,4-Dihydroxyacetophenone and 3,4-Dihydroxybenzaldehyde were procured from Spectrochem Pvt. Ltd. Chitosan (low molecular weight 50,000–190,000 g mol^−1^, viscosity 20–300 cp, 75–85% deacetylated) is purchased from Sigma Aldrich (Bengaluru, India). Ceric ammonium nitrate (CAN), potassium hydroxide, silica gel (100–200 mesh size), and petroleum ether were purchased from SRL Pvt. Ltd (Chennai, India). Methanol, ethyl acetate, acid solutions, and TLC plate (Aluminium oxide 150 F254, neutral) were acquired from Merck (Bengaluru, India). Adhesive gauze and bandages were procured from the local pharmacy. Carbon powder, polyvinylpyrrolidone (PVP), and ethylene glycol (EG) were purchased from Sigma-Aldrich. Conductive AgCl paste was procured from ELTECKS corporation (Bengaluru, India). All other chemicals and solvents were of research grade and used without further purification. Deionized (DI) water from the Millipore system with resistivity > 18.2 MΩ/cm was used throughout the experiment.

### 2.2. Preparation of Electrode Ink

CSB was synthesized using the graft polymerization method according to the previously reported process [26]. The electrode ink was prepared by dissolving CSB (5 mg) in DI water (1 mL) with Nafion (100 μL) as the binder. The viscous conductive carbon ink was achieved by the following protocols. PVP (100 mg) was dissolved in ethylene glycol (10 mL) by continuous magnetic stirring for 1 hr. Carbon powder (1 g) was added to the above solution under stirring for 10 min. The resulting viscous slurry was probe sonicated for 20 min in cold conditions.

### 2.3. Design and Fabrication of Flexible Electrode

**Figure 1:**
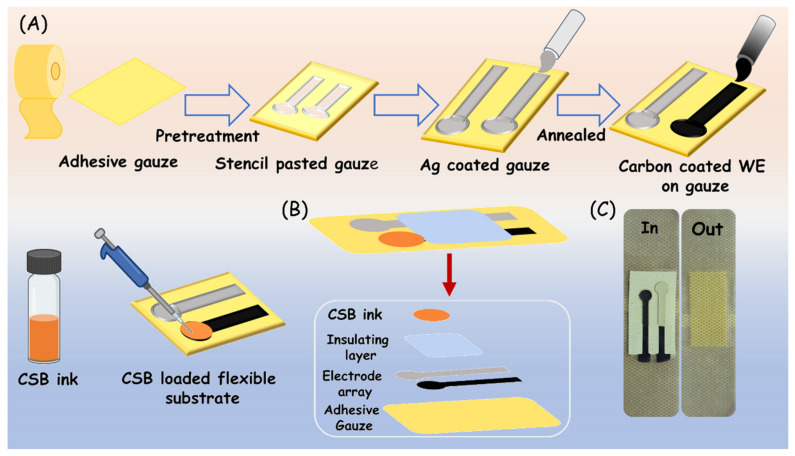
Schematic representation of fabrication of a bandage-based adhesive electrode.

Commercial wound gauze was cut down in the dimension of 2.5 × 1.5 cm^2^ (L × W). To circumvent the wicking properties of the gauze substrate, at first, it was treated with the wax solution by immersing it for 15 min. For which the paraffin (2 mg/mL) in n-hexane was prepared by ultrasonication for 10 min. The sensor pattern for the two-electrode system was designed using Corel-draw software, and the stencil for printing the electrodes was carved on flexible polypropylene sheets using a GRAPHTEC 2D cutting plotter. As-prepared stencils and the electrode ink then underwent manual screen printing. Typically, a conductive AgCl ink track was printed on the gauze and annealed at 50 °C for 1 h. Following this, the conductive carbon ink was deposited only on the working electrode area over the AgCl-coated gauze and further annealed at 50 °C to obtain a uniform coating of the electrode. The carbon-coated surface acts as a working electrode (WE) and the AgCl track as a reference electrode (RE). Finally, the CSB electrode ink was modified over the carbon-based working substrate. Figure 1 shows the steps involved in the fabrication of an adhesive two-electrode system on wound gauze and translated into a bandage system.

## 3. Results

Before constructing the CSB-loaded conductive carbon deposited on the AgCl-coated gauze (Ag/C/CSB) as a flexible electrode system, several optimizations were conducted suitable for the adhesive electrode system. For instance, to yield colloidal stability and efficient coating on the adhesive substrate, the pristine CSB ink was mixed with cellulose [29]. However, this largely impaired the conductivity of the CSB ink at the electrode interface (Figure S1A). Thus, to alter the conductivity and obtain the current response of the CSB electrode material, a conductive AgCl-coated flexible two-electrode system was constructed and evaluated their electrochemical behavior. Unpredictably AgCl peak was interfered much at the potential of +0.16 V, which also reflected from the changes in the electrode surface color, i.e., the oxidation of silver to dark brown (Figure S1B).

**Figure 2:**
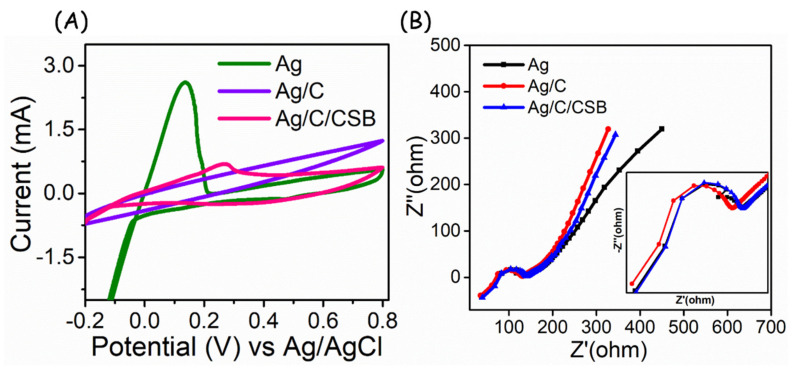
(**A**) CV response of constructed layers in CSB−based adhesive electrodes. (**B**) EIS characteristics of different layers on CSB-based adhesive electrodes.

In order to overcome the adverse effect of AgCl at the developed electrode system, the conductive carbon ink was introduced with the AgCl-coated gauze (Ag/C), followed by the loading of CSB. To quantitatively evaluate the electrochemical performance of developed Ag, Ag/C and CSB-loaded Ag/C electrodes (Ag/C/CSB), a CV analysis was performed in PBS electrolyte 0.1 M (pH-7) at a scan rate of 0.05 V/s. As can be seen in Figure 2A, the Ag electrode exhibits an anodic peak potential (*E*_pa_) at +0.16 V, whereas the Ag/C electrode exhibit the mere non-faradaic response, and the developed Ag/C/CSB electrode exhibits an *E*_pa_ at +0.25 V with an anodic peak current of 0.7 mA, suggesting the superior conductivity of integrated CSB ink. The electrochemical impedance analysis was also performed to elucidate the charge transfer resistance (R_ct_) behavior using 5 mM of K_3_[Fe(CN)_6_] and K_4_[Fe(CN)_6_] in 50 mM KCl solution (Figure 2B). The R_ct_ values were calculated to be ~130 Ω for Ag, ~141 for Ag/C, and ~142 Ag/C/CSB electrodes, suggesting the deployability of the developed adhesive bandage electrode for electrochemical signal transduction.

**Figure 3:**
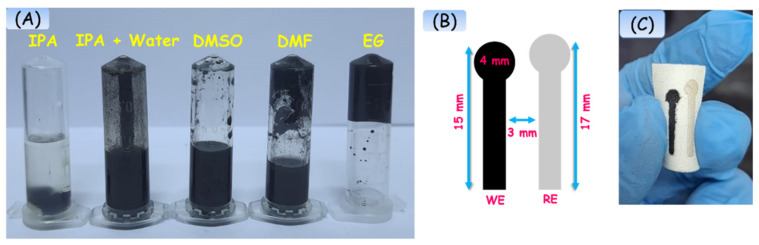
(**A**) Photograph of optimized carbon ink with various solvents. (**B**) Schematic representation of geometrical dimension of electrode designed using Corel-draw software and printed on 2D cutter plotter. (**C**) Image of the developed flexible electrode on wound gauze.

Figure 3A depicts the images of an optimized conductive carbon ink prepared with PVP as a binder dispersed in various solvents such as isopropanol (IPA), water:IPA, dimethyl sulfoxide (DMSO), dimethylformamide (DMF), and ethylene glycol (EG). Among the prepared dispersions, carbon ink with PVP and EG exhibits better colloidal stability and feasibility for coating on the gauze. Figure 3B,C illustrates the Corel-draw designed geometrical dimension and photographic image of the two-electrode systems on a wound gauze having 4 mm of WE and RE surface.

**Figure 4:**
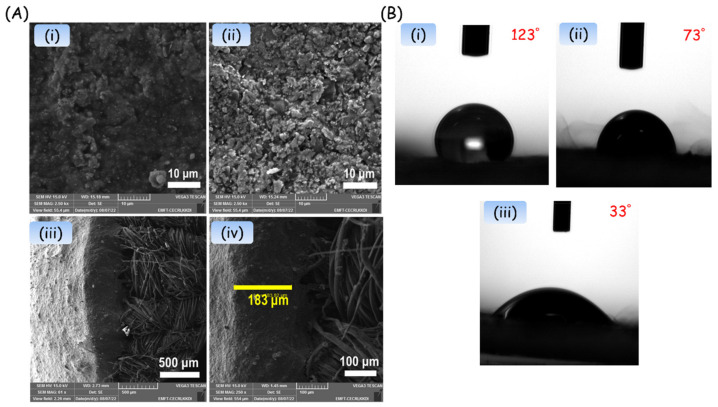
(**A**) SEM images of a flexible two-electrode system. (i) Carbon-based WE, (ii) AgCl-based RE. (iii) & (iv) represent the cross-sectional images of the electrode system. (**B**) Contact angle measurement of electrode surface (i) RE, (ii) conductive carbon, and (iii) CSB loaded WE.

Surface morphology and coating efficiency of conductive ink are essential properties of printable ink for reproducible sensor construction. Figure 4 depicts the SEM images of carbon deposited WE, AgCl-coated RE, and the cross-sectional images of the developed electrode. The WE and RE electrode surfaces (Figure 4A (i) and (ii)) exhibit a dense and homogeneous coating without cracks expected to have the desired electrical conductivity of the electrode. The cross-sectional images of the electrode (Figure 4A (iii) & (iv)) also suggest a firm coating on the fibrous surface of the gauze at the desired dimension. The coating thickness of the electrode was measured to be 183 ± 2 μm. Even after annealing, the conductive ink coated gauze substrate exhibits a homogeneous texture without pores and cracks on the electrode surface.

The wettability nature of the developed electrode surface was validated by the sessile drop contact angle measurement, which tested 3 μL water droplets on pristine WE, RE, and Ag/C/CSB electrodes (Figure 4B). As can be seen, the pristine RE surface exhibits a contact angle of 123°, evidencing the hydrophobic nature of the electrode surface. On the other hand, the pristine Ag/C shows a contact angle of 73°, which is attributed to the localized carbon ink. Similarly, upon the introduction of CSB on the Ag/C surface, the contact angle was further shifted to 33°, denoting a more hydrophilic nature of the electrode surface.

**Figure 5:**
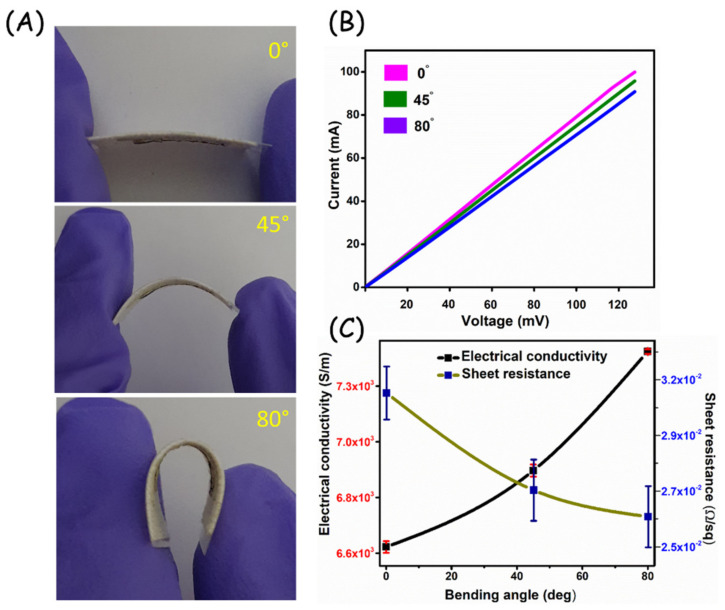
(**A**) Optical images of different bending angles of Ag/C/CSB electrode. (**B**) I−V curve of Ag/C/CSB electrode at different bending angles and (**C**) its plot of electrical conductivity and sheet resistance with twisting angles. All the experiments were performed in triplicate (*n* = 3), and the relevant error bars in the calibration plot of conductivity and sheet resistance represent the standard deviation of the mean.

To evaluate the flexible nature of the developed electrode system, a bending test was performed at different angles and measured the electrical conductivity and sheet resistance. It can be seen in Figure 5A, the images of an electrode at different bending angles and its performance in terms of the I-V curve (Figure 5B). The plot of electrical conductivity and sheet resistance at different bending angles of the Ag/C/CSB electrode system is depicted in Figure 5C. The formula used for calculating the electrical conductivity and sheet resistance is given in the supporting information. The average electrical conductivity of the developed Ag/C/CSB electrode was calculated to be 6.9 × 10^3^ S m^−1^.

**Figure 6:**
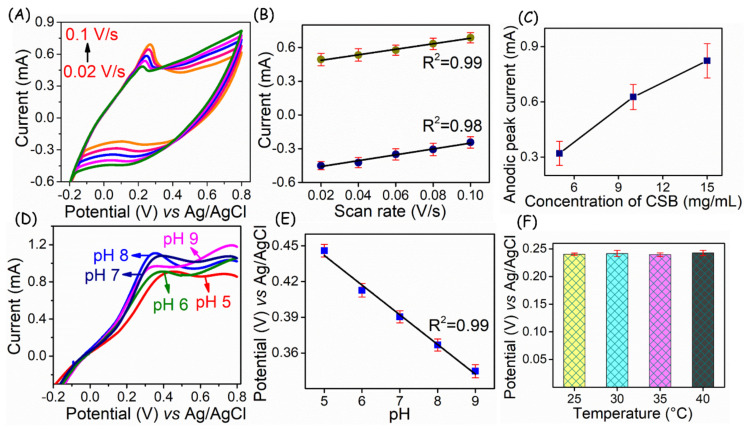
(**A**) Scan rate dependent study of CSB loaded electrodes and its linear plot (**B**). (**C**) Current response for the Ag/C/CSB electrodes at different concentrations loading of CSB. (**D**) Voltammetric changes of CSB loaded electrode in different pH solutions and its linear plot (**E**). (**F**) Histogram of Ag/C/CSB electrode performance at different annealed temperatures. All the experiments were performed in triplicate (*n* = 3).

A scan rate dependent study was performed (Figure 6A) to ensure the electrochemical process of CSB-loaded adhesive electrodes at the interfaces. As can be seen, the peak current increases with the applied scan rate and its linear plot (Figure 6B) for the scan rate vs. peak current, enabling a coefficient of variation of 0.99, indicating the surface-confined process occurs at the electrode-electrolyte interface. The electrochemical behavior of CSB is attributed to the conversion of the catecholic OH group to quinone formation, suggesting a two-proton and two-electron transfer. To verify the mobility of electron transfer at the interface, different concentrations of CSB-loaded electrodes were tested (Figure 6C). From which a sequential current increment with respect to loading was observed, suggesting their potential utility in high surface area signal transduction.

For the proof of concept, CSB-loaded flexible substrates were demonstrated for pH sensors to disseminate its potentiality in wound healing monitoring. In general, wound site pH is regarded as the key determinant for analyzing the healing status [30,31] to rationalize wound therapy and to prepare the debridement. As a result, the developed electrodes were deployed to understand the protonation and deprotonation effect in various pH conditions. The linear sweep voltammetry (LSV) technique was adopted for monitoring the potential changes under varied pH conditions in PBS (pH 5 to 9) solutions.

Unlike normal skin pH of 5–6, wound site exhibit a pH between 7 and 9 [32]. Therefore, in this work, the prepared electrodes were tested in the above pH regions. As can be seen in Figure 6D, pH analysis of Ag/C/CSB flexible electrode exhibits a distinguishable potential shift ranging from +0.44 to +0.33 V in the pH 5 to 9. The linear plot of pH vs. peak potential (Figure 6E) exhibits a better Nernstian response of −25 ± 0.3 mV pH^−1^, suggesting the fast and reliable response of Ag/C/CSB electrode surface prepared on the wound gauze. To understand the better resolution of the developed sensor, open circuit potential is measured in the pH range of 6.5 to 8.5, which is depicted in Figure S3. Further, to evaluate the sensor performance in different surface temperatures, the as-fabricated electrode was exposed to 25, 30, 40, and 50 °C. Relevant results pertaining to anodic peak potential variations of the electrodes derived from LSVs are provided in Figure 6F.

## 4. Discussion

Chronic wounds fail to undergo a natural curing process because of various physiological and environmental factors. Amongst, alteration of pH at wound sites indicates/governs the natural healing, impairment due to microbial contamination and biochemical process. A diabetic foot ulcer is one such chronic/non-healing wound having an elevated pH range from 7 to 9, whereas the normal skin pH appears to be ≤pH 6 [33]. Recent advancements in printable electrode ink show the possibilities of constructing a (bio)sensor platform for various clinical biomarkers [34,35]. In that aspect, a global challenge exists in this field, particularly in devising biocompatible yet redox-active systems for the fabrication of label-free electrochemical biosensors [36]. Herein, a CSB bioconjugate-modified wound gauze is developed for the wound site pH analysis.

As known, chitosan provides biocompatibility and flexibility for preparing wound dressing material [2]. The reactive OH group that exists on the polyphenolic butein ring of CSB yielded an additional feature to the prepared system enabling redox behavior for external mediator-free sensing applications. The CSB-loaded biomedical gauze reinforced with carbon-deposited silver matrix for monitoring the pH shows promising utility for the wound site analysis. From the electrode matrix optimization study, Ag/C/CSB composition is found to be superior in exerting conductivity convenient for rational electrochemical transduction on the adhesive bandage. Out of the studied binder-solvent (IPA, IPA:H_2_O; EG, DMSO, and DMF) ratio, PVP in combination with EG enabled the suitable condition for homogenous hydrophobicity on the wound gauze alleviating the overlay of Ag/C/CSB coating. The prepared PVP/EG composite was previously explored by researchers for wound dressing applications. For instance, Anjum et al. have developed CS/PEG/PVP hydrogel matrix for wound dressing and evaluated their biocompatibility on the repaired tissues [37]. Likewise, Tan et al. have reported CS-stabilized poly(ethylene glycol) diacrylate/2-hydroxyethyl methacrylate (PEG-DA/ HEMA) copolymer hydrogel microspheres as drug carriers for tissue engineering [38]. Thus, the usage of similar polymer blends in the Ag/C/CSB electrode will improve the biocompatibility and recognition of the immune system for wound site analysis. The working interface of the developed Ag/C/CSB electrode is hydrophilic, which might be exposed on the wound site, whereas the remaining part gauze is hydrophobized. As discussed in the above-cited literature, the ethylene oxide linkage in PEG is responsible for improving the dispersibility/hydrophilicity of the system, which is an essential requirement for absorbing the draining fluid in the wound site.

The proper structural design with the retained mechanical stability of flexible electrodes is essential for yielding good contact with the body surface. Particularly human skin undergoes stretching and bending in day-to-day activity [39]. Hence, any surface adhesive sensor substrate should be able to detect a reliable signal at different strain/stress conditions. In this work, the as-prepared sensor platform was evaluated for its performance at different stretching/bending angles. Before measuring the I-V curve analysis, the electrode underwent bending for around 10 s, and the obtained results showed a superior current flow at the surface. Variations in the conductivity and sheet resistance denote that the prepared Ag/C/CSB-based adhesive platform maintains its superior performance even at 80° of deflected angle. The fundamental CV studies of Ag/C/CSB loaded adhesive electrode in PBS electrolyte exerts redox behavior ascribed to the anodic reaction of catecholic OH group to quinone moiety, following 2e^−^/2H^+^ transfer at the electrode-electrolyte interface. Moreover, the distinguishable oxidation potential with respect to different pH suggests the inherent redox activity of CSB within the Ag/C matrix. Such pH probing transducers are known to have viable applications in monitoring the wound exudate condition. Alteration of wound site dressings and treatment is a painful process, especially with chronic illnesses such as diabetes and inflammatory conditions. This proof-of-concept demonstrated with biofriendly redox-active material on the medical gauze shows their versatile analytical performance in pH monitoring via simple voltammetric technique, promising toward wound site analysis. The wound environment is dynamic, with complex biomarkers due to various patho-physiological processes [5,31]. Thus, achieving sensitive signal transduction specific to healing protein markers is an essential topic of research. Until now, there are no embedded (bio)sensor platforms available commercially for monitoring the wound site pH and inflammatory markers. Therefore, the development of such bioadhesive substrates suitable for dual functionality would certainly bring value to advanced healthcare diagnostics.

## 5. Conclusions

In summary, a new flexible sensing platform utilizing biopolymer-derived composites was developed for monitoring pH based on the inherent potential difference applicable for assessing wound healing status. The flexible sensor substrate composed of two electrodes on the wound gauze (2.5 × 1.5 cm^2^) is translated into an adhesive system for testing the various pH. The CSB-loaded adhesive electrodes revealed their hydrophilicity, transforming their suitability for wound fluid analysis. The CSB-based modified system undergoes 2e^−^/2H^+^ transfer at the interface exerting distinguishable signal in the pH 5 to 9 with a Nernstian sensitivity of −25 ± 0.3 mV pH^−1^.Utility of such platform for real wound exudate monitoring; specifically, the healing markers still need improvisation in terms of selectivity, specificity, and sensitivity.

## Data Availability

The original contributions presented in the study are included in the article/Supplementary Materials; further inquiries can be directed to the corresponding author.

## Supplementary Materials

The following are available online at https://www.mdpi.com/article/10.3390/bios13010006/s1, Figure S1: Fabrication of CSB-coated electrodes in the flexible substrate. (A) Prepared CSB ink coated on wound gauze and its conductivity measurement. (B) Conductive AgCl ink coated OHP substrate and loading of CSB on the flexible substrate. Images of oxidized electrodes after electrochemical measurement. The formula for the electrical conductivity and sheet resistance calculation. Figure S2: Optical images of resistivity measurement of the constructed layer of Ag/C/CSB electrode. Figure S3: Open circuit potential of Ag/C/CSB electrode performance in different pH solutions and its linear plot. Reference [40] is cited in the Supplementary Materials.

## References

[1] Liang X., Li H., Dou J., Wang Q., He W., Wang C., Li D., Lin J.-M., Zhang Y. (2020). Stable and Biocompatible Carbon Nanotube Ink Mediated by Silk Protein for Printed Electronics. Adv. Mater..

[2] Kim J., Cai Z., Lee H.S., Choi G.S., Lee D.H., Jo C. (2011). Preparation and characterization of a Bacterial cellulose/Chitosan composite for potential biomedical application. J. Polym. Res..

[3] Huang Y., Song Y., Gou L., Zou Y.A.-O. (2021). A Novel Wearable Flexible Dry Electrode Based on Cowhide for ECG Measurement. Biosensors.

[4] Lv J., Thangavel G., Li Y., Xiong J., Gao D., Ciou J., Tan M.W.M., Aziz I., Chen S., Chen J. (2021). Printable elastomeric electrodes with sweat-enhanced conductivity for wearables. Sci. Adv..

[5] Gao Y., Nguyen D.T., Yeo T., Lim S.B., Tan W.X., Madden L.E., Jin L., Long J.Y.K., Aloweni F.A.B., Liew Y.J.A. (2021). A flexible multiplexed immunosensor for point-of-care in situ wound monitoring. Sci. Adv..

[6] Lagoumintzis G., Zagoriti Z., Jensen M.S., Argyrakos T., Koutsojannis C., Poulas K. (2019). Wireless Direct Microampere Current in Wound Healing: Clinical and Immunohistological Data from Two Single Case Reports. Biosensors.

[7] Yang W., Wang C. (2016). Graphene and the related conductive inks for flexible electronics. J. Mater. Chem. C.

[8] Jafarpour M., Nüesch F., Heier J., Abdolhosseinzadeh S. (2022). Functional Ink Formulation for Printing and Coating of Graphene and Other 2D Materials: Challenges and Solutions. Small Sci..

[9] Ditta N.A., Yaqub M., Nadeem S., Jamil S., Hassan S.U., Iqbal S., Javed M., Elkaeed E.B., Alshammari F.H., Alwadai N. (2022). Electrochemical Studies of LbL Films With Dawson Type Heteropolyanion Glassy Carbon Electrode Sensor Modified for Methyl Parathion Detection. Front. Mater..

[10] Khalilpour H., Shafiee P., Darbandi A., Yusuf M., Mahmoudi S., Moazzami Goudarzi Z., Mirzamohammadi S. (2021). Application of Polyoxometalate-based composites for sensor systems: A review. J. Compos. Compd..

[11] Zaumseil J. (2015). Single-walled carbon nanotube networks for flexible and printed electronics. Semicond. Sci. Technol..

[12] Menon H., Aiswarya R., Surendran K.P. (2017). Screen printable MWCNT inks for printed electronics. RSC Adv..

[13] Tian H., Tang Z., Zhuang X., Chen X., Jing X. (2012). Biodegradable synthetic polymers: Preparation, functionalization and biomedical application. Prog. Polym. Sci..

[14] Hutmacher D.W. (2000). Scaffolds in tissue engineering bone and cartilage. Biomaterials.

[15] Jia M., Kim J., Nguyen T., Duong T., Rolandi M. (2021). Natural biopolymers as proton conductors in bioelectronics. Biopolymers.

[16] Ho C.M.B., Mishra A., Lin P.T.P., Ng S.H., Yeong W.Y., Kim Y.-J., Yoon Y.-J. (2017). 3D Printed Polycaprolactone Carbon Nanotube Composite Scaffolds for Cardiac Tissue Engineering. Macromol. Biosci..

[17] Zhang J., Zhao S., Zhu M., Zhu Y., Zhang Y., Liu Z., Zhang C. (2014). 3D-printed magnetic Fe3O4/MBG/PCL composite scaffolds with multifunctionality of bone regeneration, local anticancer drug delivery and hyperthermia. J. Mater. Chem. B.

[18] Hu W., Chen S., Yang Z., Liu L., Wang H. (2011). Flexible Electrically Conductive Nanocomposite Membrane Based on Bacterial Cellulose and Polyaniline. J. Phys. Chem. B.

[19] Kai D., Prabhakaran M.P., Jin G., Ramakrishna S. (2011). Polypyrrole-contained electrospun conductive nanofibrous membranes for cardiac tissue engineering. J. Biomed. Mater. Res. Part A.

[20] Cheung R.C., Ng T.B., Wong J.H., Chan W.Y. (2015). Chitosan: An Update on Potential Biomedical and Pharmaceutical Applications. Mar. Drugs.

[21] Li X., Gao Y., Xiong H., Yang Z. (2021). The electrochemical redox mechanism and antioxidant activity of polyphenolic compounds based on inlaid multi-walled carbon nanotubes-modified graphite electrode. Open Chem..

[22] Kanagavalli P., Radhakrishnan S., Pandey G., Ravichandiran V., Perumal Pazhani G., Veerapandian M., Hegde G. (2020). Electrochemical Tracing of Butein Using Carbon Nanoparticles Interfaced Electrode Processed from Biowaste. Electroanalysis.

[23] Shan Q., Tian J., Ding Q., Wu W. (2022). Electrochemical sensor based on metal-free materials composed of graphene and graphene oxide for sensitive detection of cadmium ions in water. Mater. Chem. Phys..

[24] Gandhi M., Chen S.-S., Ray S.S., Jaiswal N.K., Ranjan S., Dasgupta N., Ranjan S., Lichtfouse E., Mishra B.N. (2021). Phyto-Nanosensors: Advancement of Phytochemicals as an Electrochemical Platform for Various Biomedical Applications. Environmental Nanotechnology Volume 5.

[25] Darshani P., Gumpu M.B., Thumpati P., Rayappan J.B.B., Ravichandiran V., Pazhani G.P., Veerapandian M. (2018). Chemically synthesized butein and butin: Optical, structure and electrochemical redox functionality at electrode interface. J. Photochem. Photobiol. B: Biol..

[26] Krishnan V., Pandey G.R., Babu K.A., Paramasivam S., Kumar S.S., Balasubramanian S., Ravichandiran V., Pazhani G.P., Veerapandian M. (2021). Chitosan grafted butein: A metal-free transducer for electrochemical genosensing of exosomal CD24. Carbohydr. Polym..

[27] Brown M.S., Ashley B., Koh A. (2018). Wearable Technology for Chronic Wound Monitoring: Current Dressings, Advancements, and Future Prospects. Front. Bioeng. Biotechnol..

[28] Bates M. (2020). The Future of Wound Care. IEEE Pulse.

[29] Kamel S., Khattab T.A. (2020). Recent Advances in Cellulose-Based Biosensors for Medical Diagnosis. Biosensors.

[30] Schreml S., Szeimies R.-M., Karrer S., Heinlin J., Landthaler M., Babilas P. (2010). The impact of the pH value on skin integrity and cutaneous wound healing. J. Eur. Acad. Dermatol. Venereol..

[31] Drinkwater S.L., Smith A., Burnand K.G. (2002). What Can Wound Fluids Tell Us About the Venous Ulcer Microenvironment?. Int. J. Lower Extrem. Wounds.

[32] RoyChoudhury S., Umasankar Y., Jaller J., Herskovitz I., Mervis J., Darwin E., Hirt P.A., Borda L.J., Lev-Tov H.A., Kirsner R. (2018). Continuous Monitoring of Wound Healing Using a Wearable Enzymatic Uric Acid Biosensor. J. Electrochem. Soc..

[33] Bennison L., Miller C., Summers R.H., Minnis A., Sussman G., McGuiness W. (2017). The pH of wounds during healing and infection: A descriptive literature review. Wound Pract. Res..

[34] Mathew M., Radhakrishnan S., Vaidyanathan A., Chakraborty B., Rout C.S. (2021). Flexible and wearable electrochemical biosensors based on two-dimensional materials: Recent developments. Anal. Bioanal. Chem..

[35] Sardini E.A.-O., Serpelloni M.A.-O., Tonello S.A.-O. (2020). Printed Electrochemical Biosensors: Opportunities and Metrological Challenges. Biosensors.

[36] Andryukov B.G., Besednova N.N., Romashko R.V., Zaporozhets T.S., Efimov T.A. (2020). Label-Free Biosensors for Laboratory-Based Diagnostics of Infections: Current Achievements and New Trends. Biosensors.

[37] Anjum S., Arora A., Alam M.S., Gupta B. (2016). Development of antimicrobial and scar preventive chitosan hydrogel wound dressings. Int. J. Pharm..

[38] Tan G., Liao J., Ning C., Zhang L. (2012). Preparation, characterization, and drug-release properties of PEG-DA-based copolymer hydrogel microspheres. J. Appl. Polym. Sci..

[39] Zhao S., Li J., Cao D., Zhang G., Li J., Li K., Yang Y., Wang W., Jin Y., Sun R. (2017). Recent Advancements in Flexible and Stretchable Electrodes for Electromechanical Sensors: Strategies, Materials, and Features. ACS Appl. Mater. Interfaces.

[40] Naftaly M., Das S., Gallop J., Pan K., Alkhalil F., Kariyapperuma D., Constant S., Ramsdale C., Hao L. (2021). Sheet Resistance Measurements of Conductive Thin Films: A Comparison of Techniques. Electronics.

