# Supplementary material for: Bioadhesive Gauze Embedded with Chitosan-Butein Bioconjugate: A Redox-Active pH Sensor Platform
Source: Biosensors (Basel). 2022 Dec 21;13(1):6. doi: 10.3390/bios13010006 (PMC9855405; doi:10.3390/bios13010006)
Supplement: Supplementary file 1 [file biosensors-13-00006-s001.zip › biosensors-2074023-supplementary.pdf]

Supplementary Material

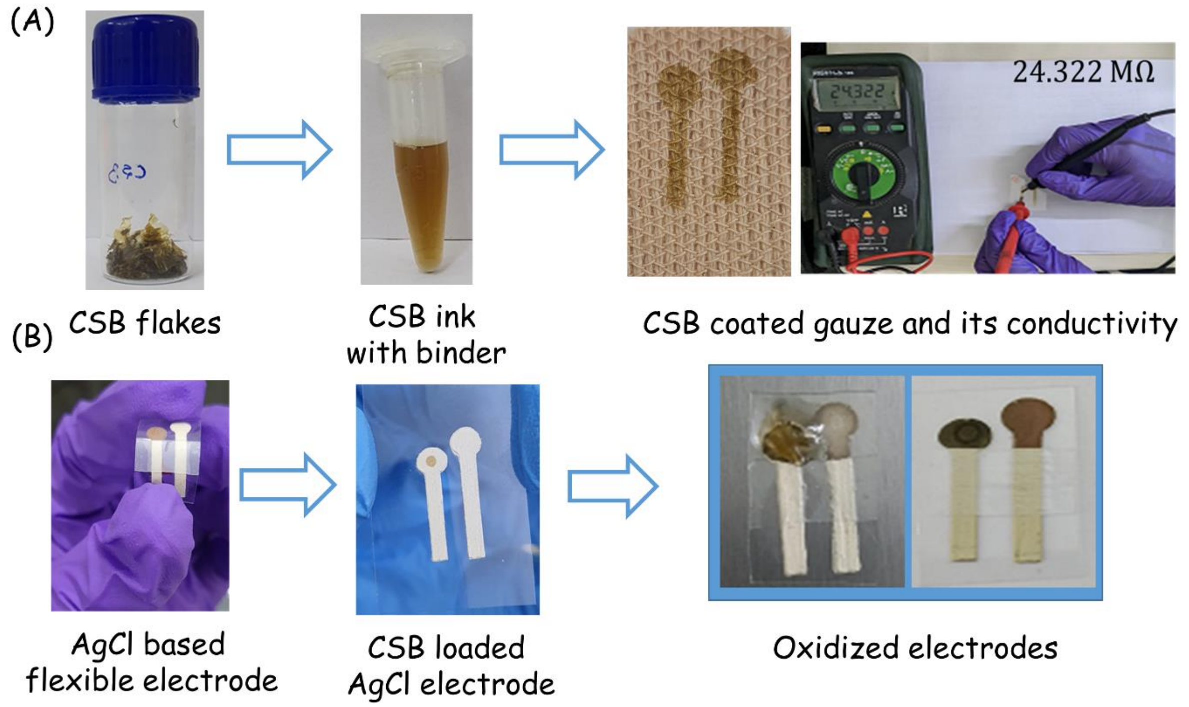

Figure S1. Fabrication of CSB coated electrodes in the flexible substrate. (A) Prepared CSB ink coated on wound gauze and its conductivity measurement. (B) Conductive AgCl ink coated OHP substrate and loading of CSB on the flexible substrate. Images of oxidized electrodes after electrochemical measurement.

Electrical conductivity and sheet resistance calculation

Electrical conductivity measurement: The electrical conductivity of the prepared electrode was calculated using the formula [1].

$$\sigma = \frac{1}{R \quad d}$$

Where σ is the electrical conductivity. R is the planar resistance and d is the thickness of the electrode. The planar resistance was derived from the slope value of I-V curve measured from keithley two-probe system. The thickness of the electrode was measured from the cross sectional images of SEM.

The sheet resistance was calculated using the formula

$$R = \rho \frac{1}{t_f}$$

Where R is the resistance, ρ is the sheet resistance and t_f is the thickness of the electrode [2].

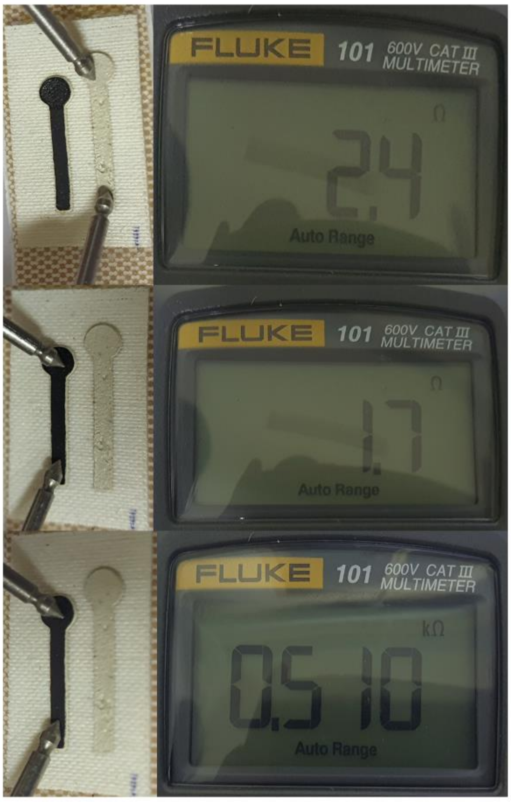

Figure S2: Optical images of resistivity measurement of the constructed layer of Ag/C/CSB electrode.

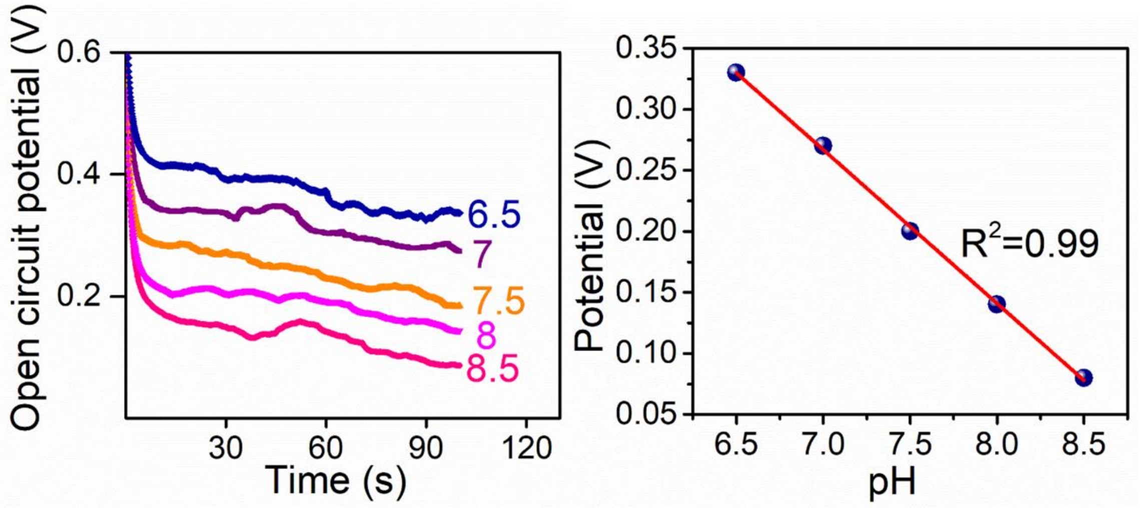

Figure S3: Open circuit potential of Ag/C/CSB electrode performance in different pH solutions and its linear plot

References

1. Liang, X.; Li, H.; Dou, J.; Wang, Q.; He, W.; Wang, C.; Li, D.; Lin, J.-M.; Zhang, Y. Stable and Biocompatible Carbon Nanotube Ink Mediated by Silk Protein for Printed Electronics. *Advanced Materials* **2020**, *32*, 2000165, doi:https://doi.org/10.1002/adma.202000165.

2. Naftaly, M.; Das, S.; Gallop, J.; Pan, K.; Alkhalil, F.; Kariyapperuma, D.; Constant, S.; Ramsdale, C.; Hao, L. Sheet Resistance Measurements of Conductive Thin Films: A Comparison of Techniques. *Electronics* **2021**, *10*, 960.
